# Transcriptional Repression of Cdc25B by IER5 Inhibits the Proliferation of Leukemic Progenitor Cells through NF-YB and p300 in Acute Myeloid Leukemia

**DOI:** 10.1371/journal.pone.0028011

**Published:** 2011-11-23

**Authors:** Satoki Nakamura, Yasuyuki Nagata, Lin Tan, Tomonari Takemura, Kiyoshi Shibata, Michio Fujie, Shinya Fujisawa, Yasutaka Tanaka, Mitsuo Toda, Reiko Makita, Kenji Tsunekawa, Manabu Yamada, Mayumi Yamaoka, Junko Yamashita, Kazunori Ohnishi, Mitsuji Yamashita

**Affiliations:** 1 Department of Internal Medicine III, Hamamatsu University School of Medicine, Higashi-ku, Hamamatsu, Shizuoka, Japan; 2 Cancer Center, Hamamatsu University School of Medicine, Higashi-ku, Hamamatsu, Shizuoka, Japan; 3 Equipment Center, Hamamatsu University School of Medicine, Higashi-ku, Hamamatsu, Shizuoka, Japan; 4 Division of Hematology, Hamamatsu Medical Center, Naka-ku, Hamamatsu, Shizuoka, Japan; 5 Department of Nano Materials, Graduate School of Science and Technology, Shizuoka University, Naka-ku, Hamamatsu City, Shizuoka, Japan; University of Turin, Italy

## Abstract

The immediately-early response gene 5 (IER5) has been reported to be induced by γ-ray irradiation and to play a role in the induction of cell death caused by radiation. We previously identified IER5 as one of the 2,3,4-tribromo-3-methyl-1-phenylphospholane 1-oxide (TMPP)-induced transcriptional responses in AML cells, using microarrays that encompassed the entire human genome. However, the biochemical pathway and mechanisms of IER5 function in regulation of the cell cycle remain unclear. In this study, we investigated the involvement of IER5 in the cell cycle and in cell proliferation of acute myeloid leukemia (AML) cells. We found that the over-expression of IER5 in AML cell lines and in AML-derived ALDH^hi^ (High Aldehyde Dehydrogenase activity)/CD34^+^ cells inhibited their proliferation compared to control cells, through induction of G2/M cell cycle arrest and a decrease in Cdc25B expression. Moreover, the over-expression of IER5 reduced colony formation of AML-derived ALDH^hi^/CD34^+^ cells due to a decrease in Cdc25B expression. In addition, over-expression of Cdc25B restored TMPP inhibitory effects on colony formation in IER5-suppressed AML-derived ALDH^hi^/CD34^+^ cells. Furthermore, the IER5 reduced *Cdc25B* mRNA expression through direct binding to *Cdc25B* promoter and mediated its transcriptional attenuation through NF-YB and p300 transcriptinal factors. In summary, we found that transcriptional repression mediated by IER5 regulates Cdc25B expression levels via the release of NF-YB and p300 in AML-derived ALDH^hi^/CD34^+^ cells, resulting in inhibition of AML progenitor cell proliferation through modulation of cell cycle. Thus, the induction of IER5 expression represents an attractive target for AML therapy.

## Introduction

Acute myeloid leukemia (AML) is characterized by the excess production of leukemic blasts arrested at various stages of granulocytic and monocytic differentiation. To effectively cure a patient with AML, this proliferation of leukemic cells must be halted. Given that chemotherapy rarely eradicates the leukemic clones, efforts are now being made to find innovative new therapies which inhibit the proliferation of AML cells. However, the effect of cell cycle progression and apoptosis resistance on the pathogenesis of AML remains to be defined. Against these backgrounds, we have synthesized new bioactive agents and then investigated these anti-leukemic effects. We previously reported that the phospha sugar derivative, 2,3,4-tribromo-3-methyl-1-phenylphospholane 1-oxide (TMPP), was synthesized in the reaction of 3-methyl-1-phenyl-2-phospholene 1-oxide with bromine, and we investigated the potential of TMPP as an anti-leukemic agent using AML-derived ALDH^hi^ cells [1]. This agent induced a G2/M cell cycle block through a reduction in cell cycle progression signals (FOXM1, KIS, Cdc25B, Cyclin D1, Cyclin A, and Aurora-B), resulting in inhibition of leukemia cell proliferation [1]. We also observed that down-regulation of FOXM1 inhibited proliferation, and demonstrated that TMPP suppressed FOXM1 expression, and that this FOXM1 repression reduced *Cyclin B1* and *Cdc25B* mRNA expression, resulting in inhibition of the proliferation of AML-derived ALDH^hi^ cells [2]. Thus, we demonstrated that TMPP-mediated FOXM1 repression induced G2/M cell cycle arrest through a reduction in Cyclin B1 and Cdc25B expression. However, TMPP and FOXM1 regulate many mitotic regulators in AML cells. It is unclear how TMPP predominantly induces G2/M cell cycle arrest rather than G1 cell cycle arrest in AML cells.

To identify TMPP-induced transcriptional responses in AML cells, TMPP-induced transcriptional alterations were investigated using microarrays that encompassed the entire human genome. About 180 genes, which belong to functional categories such as the DNA damage response, regulation of cell cycle and cell proliferation, and signaling pathways, responded to TMPP treatment at the transcriptional level in AML cells. Of these genes, the immediate-early response gene 5 (*IER5*) was identified as a key regulator of the G2/M cell cycle transition.

The immediate-early genes (*IER*), which are rapidly induced by growth factors or other various stimuli, encompass a variety of different protein families (Fos and Jun family of transcriptional regulators; Myc; zinc-finger proteins; secreted cytokines; cytoplasmic proteins, and integral membrane proteins) [3]. Activation of IER is an important initial step in the regulation of cellular and genomic responses to external stimuli. Approximately 100 *IER* genes have been described to date, and are subdivided into two classes (fast-kinetics and slow-kinetics) based on their activation kinetics [4]. The fast-kinetics *IER* genes (e.g., *c-Fos*) contain serum response elements (SRE), which are required for transcriptional induction. In contrast, the slow-kinetics *IER* genes, which lack SRE, display a relatively slower induction and longer persistence profile following stimulation compared with the fast-kinetics *IER* genes [5]. The *IER5* gene, which has been identified as a member of the *IER* gene family, belongs to the slow-kinetics *IER* genes, and is rapidly induced by stimulation with serum or with the growth factors FGF or PDGF [6]. It has been also reported that *IER5* mRNA is induced in the cerebral cortex of rats during waking and sleep deprivation [7], or in the brains of mouse embryos exposed to teratogenic valpronic acid (VPA) [8]. The *IRE5* mRNA was induced within 30 min after serum-exposure and at least 180 min after the serum-stimulation**,** but its expression was not inhibited by cycloheximide [6]. *IER5* is also upregulated by ionizing radiation at doses ranging from 0.02 to 10 Gy in lymphoblastoid AHH-1 cells [9], [10]. Moreover, it has been reported that suppression of IER5 increased HeLa cell proliferation, mitigated the inhibition of proliferation imposed by irradiation, and potentiated radiation-induced arrest at the G2/M transition [11]. These results demonstrated that IER5 expression plays an important role in radiation-mediated cell death and cell cycle checkpoints.

It has been reported that inhibition of cell proliferation in AML cells is associated with a decrease in the expression of the Cdc25B phosphatase [12], and that this phosphatase participates in G2/M checkpoint recovery and its expression is upregulated in acute myeloid leukemia cells [13]. Therefore, depletion of Cdc25B might be expected to strongly induce G2/M cell cycle arrest in AML cells. As previously reported, siRNA-mediated depletion of FOXM1 expression, or TMPP treatment, induced G2/M cell cycle arrest and inhibited AML cell proliferation through a decrease in protein expression of mitotic regulators such as Cdc25B in AML cells [1], [2]. However, it is unclear how Cdc25B expression is regulated.

In this study, we investigated the function of IER5 in leukemia cell proliferation. We found that IER5 expression inhibited the proliferation of both leukemia cell lines and of leukemic blast cells derived from AML through the transcriptional repression of Cdc25B.

## Results

### The expression of IER5 mRNA in acute myeloid leukemia cells

**Figure 1:**
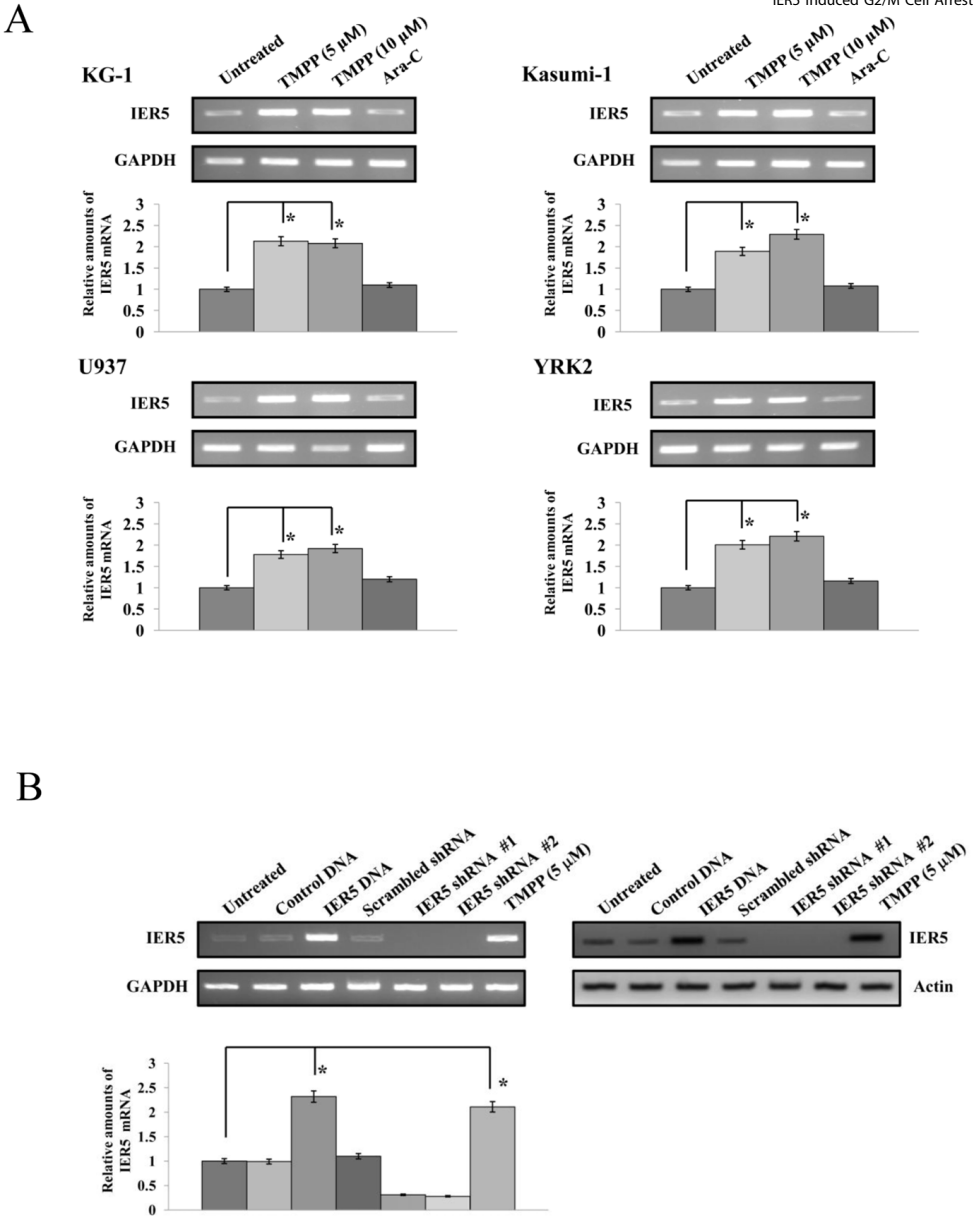
*IER5* mRNA and protein expression in AML cells. A. RT-PCR was performed to analyze *IER5* mRNA expression in AML cell lines (KG-1, Kasumi-1, U937, and YRK2) treated with or without TMPP (5 and 10 µM) or Ara-C (1 µM) for 24 h. *GAPDH* mRNA expression is shown as an internal control. RT-PCR results representative of three independent experiments are shown. Relative levels of *IER5* mRNA expression in AMLs were measured using QRT-PCR. B. *IER5* mRNA and protein expression in U937 cells that were transfected with *IER5* cDNA, shRNA-#1, or -#2, or were treated with TMPP (5 µM), were assessed using RT-PCR and QRT-PCR, and Western blotting, respectively. Actin was immunoblotted as a loading control. The expression levels of the target mRNAs were normalized to the expression of *GAPDH* mRNA. The results are expressed relative to the untreated control which was set at 1. Each RT-PCR assay was performed at least three times, and the results are expressed as means ± SD. *P<0.01. Western blotting results representative of three independent experiments are shown.

We first determined the relative expression of *IER5* mRNA in the leukemia cell lines KG-1, Kasumi-1, U937 and YRK2. As shown in Fig. 1, *IER5* mRNA was constitutively expressed in these AML cell lines. Interestingly, we found that the mRNA expression of *IER5* increased in these AML cell lines compared to untreated cells, when treated with TMPP (5 and 10 µM) for 24 h. However, Ara-C (1 µM) did not affect *IER5* mRNA expression in these AML cell lines. Furthermore, we quantified the level of *IER5* mRNA in the AML cell lines using quantitative RT-PCR. This analysis indicated that the expression of *IER5* mRNA in the TMPP-treated leukemia cells was increased 1.78 to 2.29-fold relative to its expression in untreated cells.

We next determined the relative expression levels of *IER5* mRNA and protein in U937 cells. As shown in Fig. 1B, both *IER5* mRNA and protein in U937 cells were overexpressed following transfection with *IER5* cDNA or treatment with TMPP compared to untransfected and untreated U937 cells, respectively. In contrast, transfection with *IER5* shRNA-#1 or -#2 decreased the expression of both *IER5* mRNA and protein in U937 cells compared to untransfected U937 cells. Moreover, the mRNA and protein expression of *IER5* in other AML cell lines (KG-1, Kasumi-1 and YRK2) was similarly increased by *IER5* cDNA transfection and suppressed by *IER5* shRNA transfection (data not shown). Thus, *IER5* mRNA and protein were constitutively expressed in all four AML cell lines, although their expression in AML cell lines were at lower levels compared to normal ALDH^hi^/CD34^+^ cells (data not shown). Treatment with TMPP increased both the mRNA and the protein level of IER5 in AML cells.

### Effects of IER5 expression on AML cell proliferation

**Figure 2:**
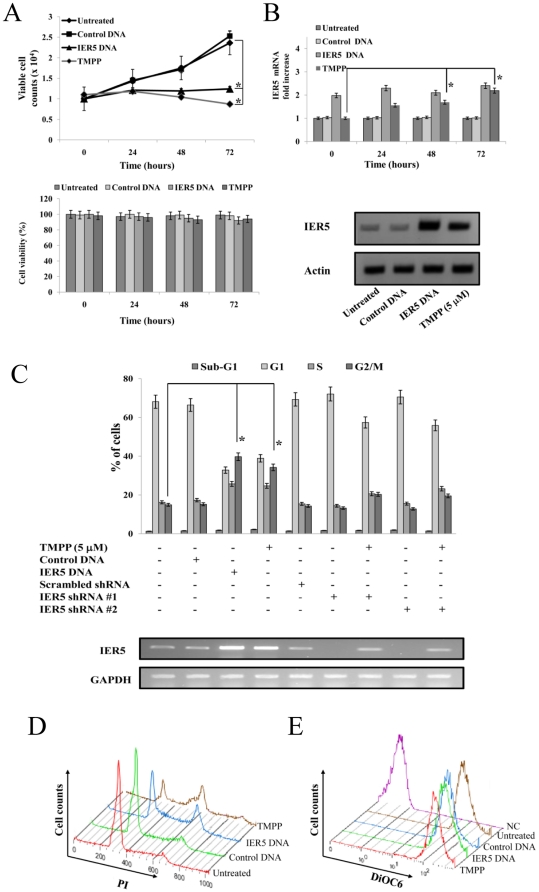
Over-expression of IER5 inhibited AML cell proliferation. A. The cell proliferation of U937 cells, transfected with IER5 cDNA or treated with TMPP, was measured by counting cells using a hemocytometer (upper panel). Cell counting was started 3 days after transfection, and was performed every 24 h for 3 days. Data are shown as means ± S.D. of triplicate cultures and are representative of three independent experiments. *P<0.01 compared with untransfected control cells. Cell viability of the IER5 over-expressing U937 cells was assessed by counting of viable cells using trypan blue staining at 72 h, starting 3 days after DNA transfection (bottom panel). B. QRT-PCR analysis of *IER5* mRNA expression in untreated cells, *IER5* cDNA-transfected cells, and TMPP-treated U937 cells. QRT-PCR was started 3 days after transfection, and was performed every 24 h for 3 days. Data are shown as means ± S.D. of triplicate cultures and are representative of three independent experiments. The levels of the quantified RT-PCR products were normalized t*o GAPDH* expression in the same sample and were then expressed relative to the mRNA level of a normal control, which was assigned a value of 1. *P<0.01 compared with untransfected control cells. The protein expression of IER5 in cells was analyzed after 3 days of culture (bottom panels). Blotting of Actin was used as a loading control. C. The cell cycle distribution of U937 cells that were transfected with control DNA, *IER5* cDNA, scrambled shRNA, IER5 shRNA #1, IER5 shRNA #1, or were treated with TMPP, was analyzed using flow cytometric analysis. The transfected or TMPP-treated U937 cells were harvested after 3 days. The fraction of cells in the G1, S and G2/M stage of the cell cycle was determined. Data are shown as means ± S.D. of triplicate cultures. The *IER5* mRNA expression of the cells is shown at bottom and was assessed using RT-PCR. The RT-PCR results are representative of three independent experiments. Data are shown as means ± S.D. of triplicate cultures. *P<0.01 compared with control cells. D and E. Cell cycle analysis (D) and changes of mitochondrial membrane potential (ΔΨm) (E) in IER5 overexpressed or TMPP treated AML cells. U937 cells were transfected with *IER5* cDNA or treated with TMPP (5 µM). Mitochondrial membrane potential was determined 3 days after transfection or TMPP treatment by staining of the cells with DiOC6 followed by flow cytometric analysis. The FACS results are representative of three independent experiments. NC; Negative control.

Since IER5 expression was induced by TMPP in AML cells, we next examined the functional importance of IER5 expression. For this purpose, we transfected U937 cells with *IER5* cDNA, and assessed the effect of IER5 over-expression on AML cell proliferation over 72 h of culture, starting from day 3 post-transfection. Cell proliferation was measured by cell counting using a hemocytometer (Fig. 2A, upper panel). When U937 cells were transfected with *IER5* cDNA, cell proliferation was inhibited compared to non-transfected control cells. Treatment with TMPP (5 µM) also significantly inhibited the proliferation of U937 cells. However, the viability of AML cells transfected with *IER5* cDNA or treated with TMPP was almost same as that of the untreated cells (Fig. 2A, bottom panel). In addition, the expression of *IER5* mRNA was significantly increased by TMPP treatment compared to untreated cells (Fig. 2B, upper panel). We further examined the effect of IER5 induction on AML cell proliferation by flow cytometric analysis of the effect of IER5 on cell cycle distribution. As shown in Fig. 2C and 2D, analysis of U937 cells on day 3 post cDNA-transfection or post-TMPP treatment indicated an increase in the percentage of cells in S and G2/M, but not in sub-G1, compared to control DNA-transfected and untreated cells. No increase in the polyploid (>4N) population of these cells was observed. On the other hand, when U937 cells were transfected with *IER5* shRNA-#1 or -#2, each cell cycle distribution was not affected compared to scrambled shRNA-transfected cells. In contrast, the population of G2/M was significantly reduced following TMPP treatment. Moreover, we determined the effect of IER5 over-expression and TMPP treatment on markers of apoptosis. We analyzed the effects on loss of mitochondrial membrane potential, which was determined by flow cytometric analysis of DiOC6 uptake. Loss of mitochondrial membrane potential is known to occur in apoptotic cells and precedes the activation of caspases. DiOC6 fluorescence of U937 cells was not reduced 3 days after transfection with *IER5* cDNA or TMPP treatment, and DiOC6 fluorescence of *IER5* cDNA-transfected or TMPP treated cells compared to control cells was observed in the almost same levels (Fig. 2E). The IER5 over-expression and TMPP treatment similarly induced G2/M cell cycle arrest, but not apoptosis in other AML cell lines (KG-1, Kasumi-1 and YRK2) (data not shown). These results showed that induction of IER5 induced a block in the G2/M phase in AML cells, but did not induce apoptosis, and therefore demonstrated that induction of IER5 expression strongly inhibited AML cell proliferation.

### Effects of IER5 knock-down on AML cell proliferation

**Figure 3:**
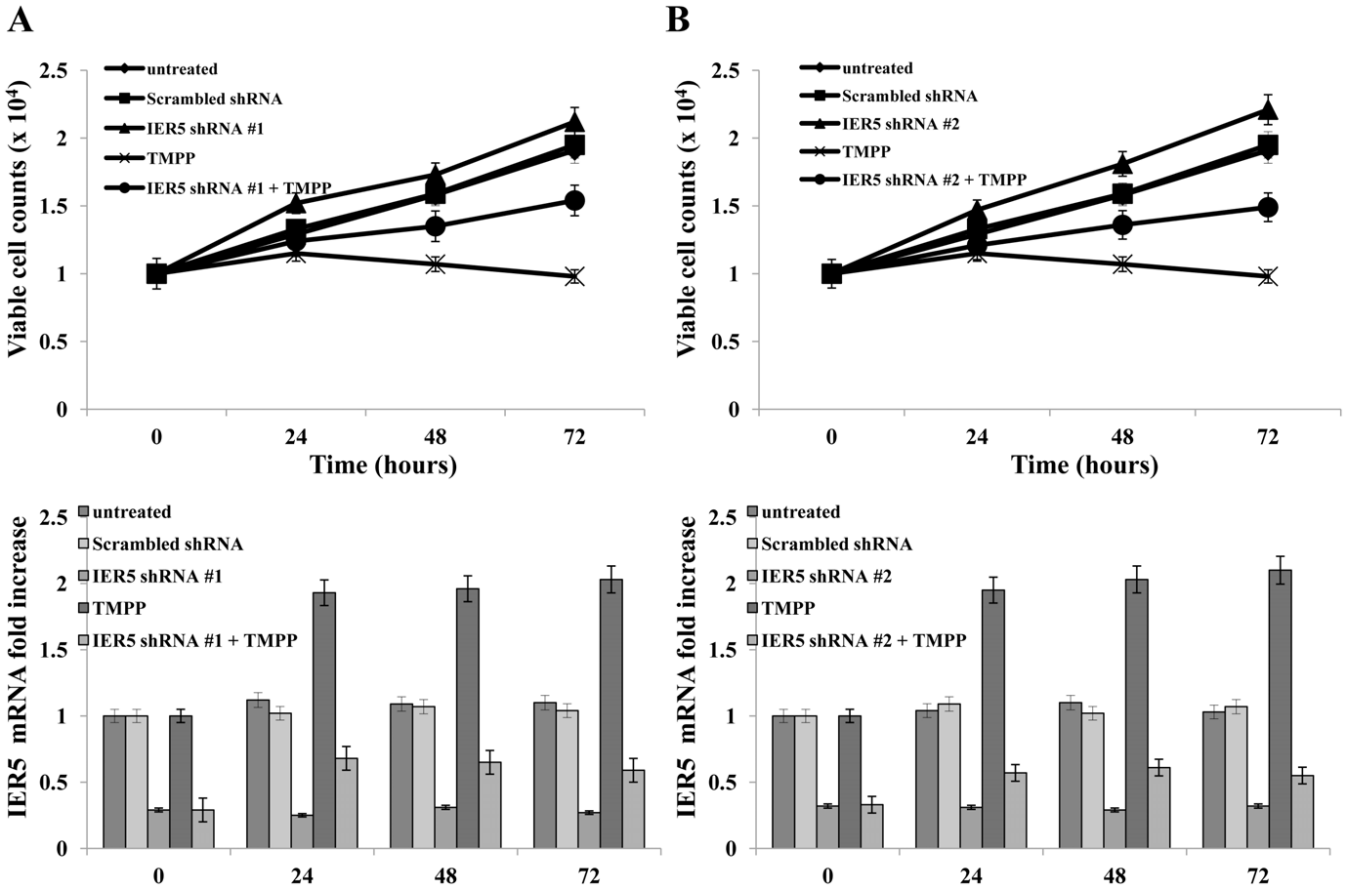
Effect of *IER5* knock-down on AML cell proliferation. U937 cells were transfected with *IER5* shRNA-#1 (A) or -#2 (B). After 3 days, the cells were treated with TMPP (5 µM) for 24, 48 and 72 h and the number of viable cells was counted (upper panels). The *IER5* mRNA level was analyzed using quantitative RT-PCR and the level was determined relative to that of *GAPDH* (bottom panels). The results are means ± SD from three independent experiments.

To further examine the functional importance of IER5 expression in AML cells, we next examined the effect of IER5 knock-down on AML cell proliferation. U937 cells were transfected with *IER5* shRNA-#1 or -#2, and the effect of IER5 knock-down on AML cell proliferation was assessed over 72 h of culture, starting from day 3 day post-transfection (Fig. 3). Cell proliferation was measured by counting the cells using a hemocytometer (upper panels). When U937 cells were transfected with *IER5* shRNA-#1 or -#2 (Fig. 3A and 3B, respectively), cell proliferation was slightly increased compared to both untreated cells and to scrambled shRNA-transfected cells. In contrast, cell proliferation was significantly reduced following TMPP treatment. However, TMPP-mediated inhibition of the proliferation of U937 cells was reduced by knock-down of IER5 via transfection of *IER5* shRNA-#1 or -#2. Moreover, TMPP-mediated IER5 induction was decreased in U937 cells transfected with *IER5* shRNA-#1 or -#2 (bottom panels) compared to non-transfected cells. Knock-down of IER5 in other AML cell lines (KG-1, Kasumi-1 and YRK2), similarly increased cell proliferation (data not shown). These results show that TMPP inhibits AML cell proliferation through IER5 induction.

### IER5 induced cell cycle arrest in AML cells through direct binding to Cdc25B promoter and decrease of NF-YB binding

**Figure 4:**
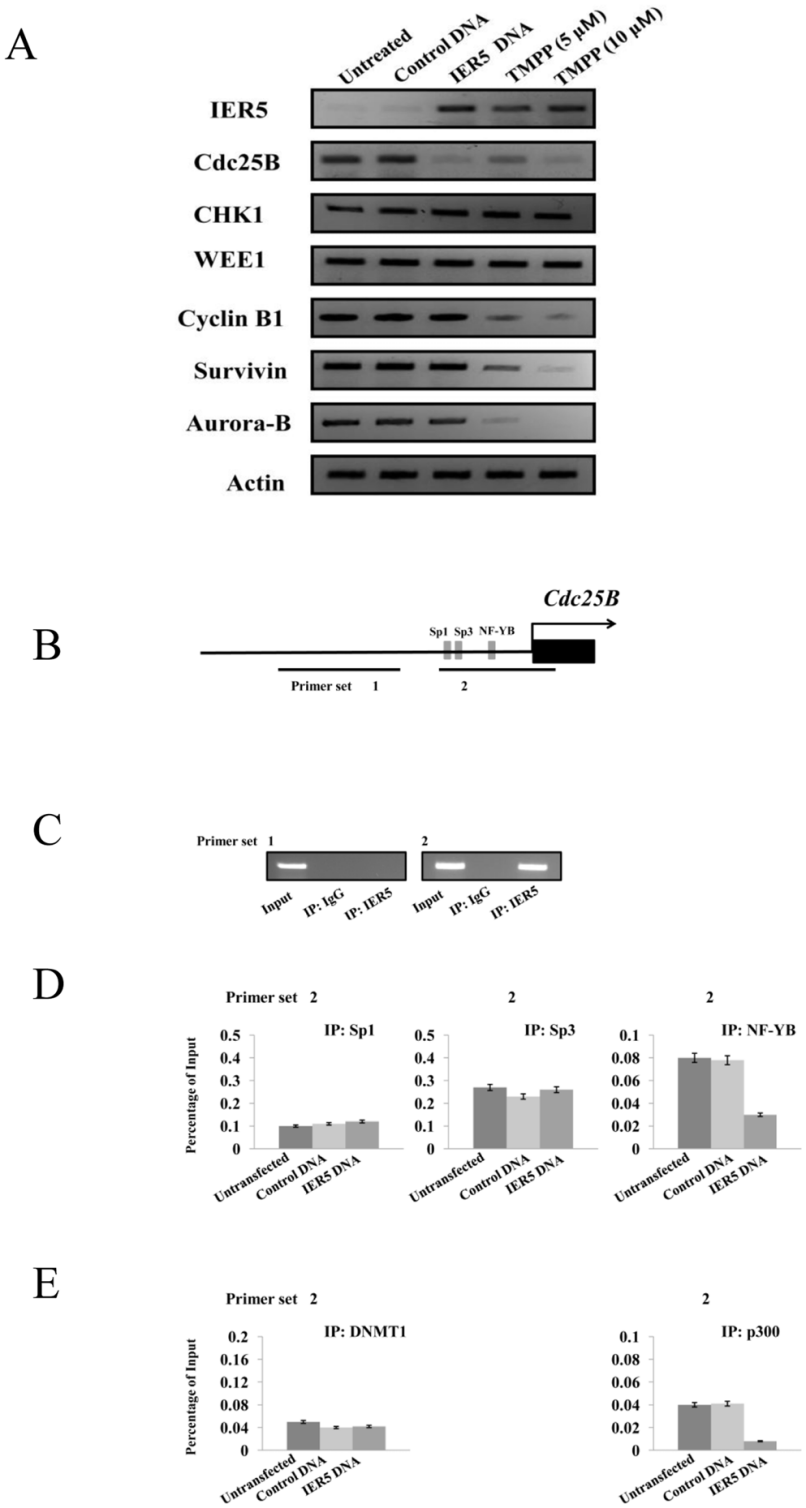
IER5 induced G2/M cell cycle arrest through direct binding to Cdc25B promoter in AML cells. A. U937 cells were transfected with control DNA, *IER5* cDNA, or were treated with TMPP (5 µM or 10 µM). The cells were harvested after 3 days, and the expression of the indicated cell cycle regulating proteins was analyzed by SDS-PAGE followed by Western blotting using the indicated specific antibodies. Blotting of Actin was used as a loading control. B. Schematic representation of the human *Cdc25B* promoter regions, indicating sites for Sp1, Sp3, and NF-YB motifs, amplified from the precipitated DNA by specific primer sets 1 and 2. C. ChIP analysis for IER5 in the *Cdc25B* promoter. D & E. IER5-dependent changes in Sp1, Sp3 and NF-YB binding (E), and the bindings of coactivators, DNMT1 and p300, to interact with Sp1 and NF-YB, respectively (F) in *Cdc25B* promoter. U937 cells were either untransfected or were transfected with the control DNA or *IER5* DNA. After 3 days culture, cells were cross-linked with 1% formaldehyde and ChIP was performed with either control antibody (IgG) or the anti-IER5 antibody. The precipitated DNA was then assayed by PCR using pairs (primer set 1 and 2) of oligonucleotides encompassing specific regions of the *Cdc25B* promoter. The values of ChIP efficiencies are given as % of input.

We next determined if the inhibitory effect of IER5 induction on cell proliferation was mediated by modulation of the expression of cell cycle regulators such as Cdc25B, CHK1, WEE1, Cyclin B1, Survivin, KIS, or Aurora B kinase, during the G2/M transition. As shown in Fig. 4A, induction of the IER5 protein in *IER5* cDNA-transfected or in TMPP-treated U937 cells was accompanied by a significant decrease in the level of Cdc25B protein compared to untransfected cells. IER5 over-expression did not affect CHK1, WEE1, Cyclin B1, Survivin or Aurora B kinase expression. In contrast, TMPP treatment reduced the expression of Cyclin B1, Survivin, and Aurora B kinase. TMPP treatment did not affect CHK1 and WEE1 expression. Induction of IER5 similarly reduced Cdc25B expression in other AML cell lines (KG-1, Kasumi-1 and YRK2) (data not shown). These results indicate that, during TMPP-mediated G2/M cell cycle arrest, TMPP reduced Cdc25B expression through IER5 induction and that IER5 is essential for the repression of Cdc25B protein expression in AML cells. Moreover, IER5 only regulated Cdc25B, and did not affect other cell cycle regulators of the G2/M transition.

To confirm binding of IER5 to the *Cdc25B* promoter and more accurately define its binding site, we performed ChIP analysis in AML cells using two pairs of oligonucleotides within upstream from the transcription start site for *Cdc25B* (Fig. 4B). ChIP analysis showed that IER5 is highly enriched within a region of the promoter of *Cdc25B*, indicating that the *Cdc25B* gene is a target for IER5 (Fig. 4C). Moreover, to determine whether the over-expression of IER5 corresponded to the binding sites of Sp1, Sp3, and NF-YB within the *Cdc25B* promoter, ChIP analysis was performed using antibodies corresponding to Sp1, Sp3, and NF-YB, which has been reported to be related to p53-mediated negative regulation of Cdc25B [14]. As shown in Fig. 4D, the reduced binding of NF-YB on the *Cdc25b* promoter was observed in IER5 over-expressed U937 cells compared to untreated and control DNA transfected cells. On the other hand, the levels of binding Sp1 and Sp3 on the *Cdc25B* promoter was not affected in untreated, control DNA-, or *IER5*-transfected cells. In addition, we investigated whether the reduction of NF-YB binding to *Cdc25B* promoter interferes with the recruitment of coactivator p300, which is anti-histone acetyltransferase as a coactivator known interact with NF-Y [15]. When U937 cell was untreated or transfected with control DNA, p300 was bound on the Cdc25B promoter. When IER5 over-expressed in U937 cells, a significant release of p300 was observed (Fig. 4E). On the other hand, the DNA methyltransferase DNMT1, which acts as a loading platform for repressor complexes such as the p53-Sp1 complexes [16] was not recruited on the *Cdc25B* promoter in *IER5* over-expressed U937 cells. These ChIP experiments show that the binding of IER5 on the *Cdc25B* promoter induced the release of the coactivator p300, but not the recruitment of the coactivator DNMT1 via Sp1 interaction, causing the down-regulation of *Cdc25B* expression.

### Induction of IER5 inhibited colony formation of AML derived-ALDH^hi^/CD34^+^ cells

**Figure 5:**
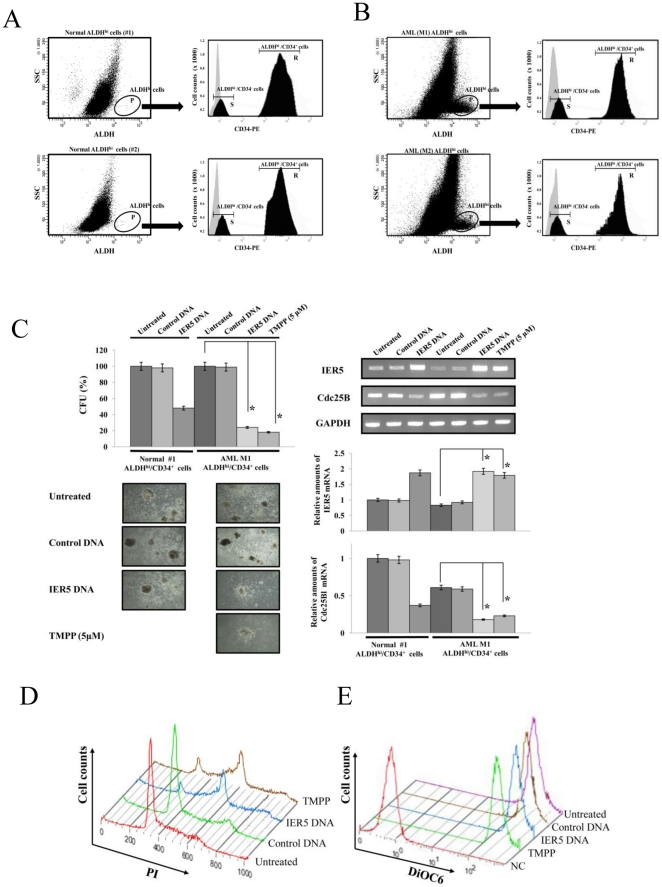
IER5 inhibits the colony formation of ALDH^hi^/CD34^+^ cells isolated from AML patients. A. Selection of ALDH^hi^/CD34^+^ hematopoietic progenitor cells from the bone marrow of two healthy volunteers (#1 and #2) by FACS sorting. Region P denotes populations of ALDH^hi^ cells. Region R and S denote populations of CD34^+^ and CD34^-^ cells in the ALDH^hi^ population (Region P), respectively. Negative control, light grey region; CD34-PE staining, dark grey region. B. Selection of ALDH^hi^/CD34^+^ hematopoietic progenitor cells from the bone marrow of two AML patients (M1 and M2) by FACS sorting. Region P denotes populations of ALDH^hi^ cells. Region R and S denote populations CD34^+^ and CD34^-^ cells in the ALDH^hi^ population (Region P), respectively. Negative control, light grey region; CD34-PE staining, dark grey region. C. ALDH^hi^/CD34^+^ cells were purified from a healthy volunteer (#1) and an AML patient (M1), and were cultured in semisolid methylcellulose media. The ALDH^hi^/CD34^+^ cells from each source were left untransfected or were transfected with *IER5* cDNA, or treated with TMPP (5 µM). After 14 days culture, the colony forming ability of the cells was analyzed (left upper panel) and the cells were viewed using phase-contrast microscopy. Original magnification ×4 (left bottom panels). Their mRNA expression of *IER5* and *Cdc25B* was assessed using RT-PCR and quantitative RT-PCR (right panels). Colonies formed by these ALDH^hi^/CD34^+^ cells (3×10^2^ to 5×10^2^ cells/plate) were counted following plating in semisolid methylcellulose media. Colony formation was evaluated by determination of colony counts as a percentage of the corresponding control. The results are the means ± SD of three independent experiments. *P<0.01 compared with untreated control cells. RT-PCR results representative of three independent experiments are shown. *GAPDH* mRNA expression is shown as an internal control. The ALDH^hi^/CD34^+^ cells whose *IER5* mRNA expression was analyzed by quantitative RT-PCR were derived from an AML patient (M1). The levels of the quantified RT-PCR products were normalized t*o GAPDH* expression in the same sample and were then expressed relative to the mRNA level of a normal control which was assigned a value of 1. D and E. Cell cycle analysis (D) and changes of mitochondrial membrane potential (ΔΨm) (E) in IER5 overexpressed or TMPP treated AML-derived ALDH^hi^/CD34^+^ cells. ALDH^hi^/CD34^+^ cells were purified from an AML patient (M1), and were then transfected with *IER5* cDNA or were treated with TMPP (5 µM). The IER5-transfected or TMPP-treated cells were harvested after 3 days. The cell cycle distribution and the ΔΨm of the ALDH^hi^/CD34^+^ cells was analyzed using flow cytometric analysis. The FACS results are representative of three independent experiments. NC; Negative control.

We next examined *IER5* mRNA expression in clinical specimens from AML patients. Hematopoietic progenitor cells from bone marrow were obtained using flow cytometry based on ALDH activity, using the substrate Aldefluor as previously reported. The CD34^+^ progenitor cells in the ALDH^hi^ population (ALDH^hi^/CD34^+^ cells) were then sorted using FACS. We isolated ALDH^hi^/CD34^+^ cells from bone marrow cells derived from 2 healthy volunteers (#1 and #2) and 2 AML patients (M1 and M2) (Fig. 5A and B). The percentage of bone marrow cells derived from healthy volunteers #1 and #2 that were ALDH^low^ was 98.6% and 97.1%, and that of ALDH^hi^ cells was 0.37% and 0.52%, respectively. The percentage of the ALDH^hi^ cells that was CD34^+^ was 84.2% and 81.9%, and that was CD34^-^ was 14.7% and 17.5%, respectively. On the other hand, in bone marrow cells derived from AML patients M1 and M2, the percentage of ALDH^low^ cells was 85.4% and 81.1%, respectively, and that of ALDH^hi^ cells was 7.4% and 6.7%, respectively. The percentage of the ALDH^hi^ cells that was CD34^+^ was 87.1% and 80.6%, and that was CD34^-^ was 11.4% and 19.3%, respectively.

To confirm that IER5 also modulated the proliferation of AML progenitor cells from patients, we analyzed the effect of IER5 expression on colony formation of ALDH^hi^/CD34^+^ cells from normal healthy volunteer #1 and from an AML patient (M1) prior to treatment (Fig. 5C). Transfection with *IER5* cDNA or treatment with TMPP induced dramatic decreases in the number of colonies formed by AML-derived ALDH^hi^/CD34^+^ cells compared to the number in untreated cells (Fig. 5C; left panels). In untransfected AML (M1) cells, the mean number of colonies was 112 (range, 103–119). Following transfection with IER5 cDNA and treatment with TMPP the mean number of colonies was 27 (range, 21–35) and 16 (range, 12–27), respectively. In contrast, transfection with IER5 cDNA only moderately decreased the number of colonies formed by normal ALDH^hi^/CD34^+^ cells, compared to untransfected normal ALDH^hi^/CD34^+^ cells. Thus, the mean number of colonies in untransfected cells was 46 (range, 39–52) but that in cells transfected with IER5 cDNA was 22 (range, 18–29). These results demonstrate that induction of *IER5* expression predominantly inhibited the proliferation of AML-derived ALDH^hi^/CD34^+^ cells. In addition, to confirm that IER5-mediated-inhibition of colony formation of AML-derived ALDH^hi^/CD34^+^ cells from patients was mediated by an effect on transcription of the IER5 target gene, we assayed the mRNA expression of the IER5 target gene *Cdc25B* in AML-derived ALDH^hi^/CD34^+^ cells. As shown in Fig. 5C (right panels), induction of IER5 expression significantly decreased the expression of *Cdc25B* mRNA in AML-derived ALDH^hi^/CD34^+^ cells, and also decreased the expression of *Cdc25B* mRNA in normal ALDH^hi^/CD34^+^ cells. Moreover, in AML (M1) ALDH^hi^/CD34^+^ cells, IER5 over-expression or TMPP treatment indicated an increase in the percentage of cells in S and G2/M compared to control cells (Fig 5D). No increase in the sub-G1 population of these cells was observed. Interestingly, we also detected an increase (3.1 to 4.2%) in the percentage of polyploid cells (>4N) in TMPP treated cells compared to control cells. In addition, as shown in Fig. 5E, DiOC6 fluorescence of AML (M1) ALDH^hi^/CD34^+^ cells was not reduced 3 days after transfection with *IER5* cDNA or TMPP treatment, and DiOC6 fluorescence of *IER5* cDNA-transfected or TMPP treated cells compared to control cells was observed in the almost same levels. Similar results were obtained using other AML ALDH^hi^/CD34^+^ cells. (data not shown). These findings suggest that the induction of IER5 expression strongly inhibited colony formation and G2/M cell cycle arrest in AML-derived ALDH^hi^/CD34^+^ cells compared to normal ALDH^hi^/CD34^+^ cells through induction of *Cdc25B* expression.

Colony forming activity of ALDH^hi^/CD34^+^ cell populations from AML patients following the induction or depletion of IER5 expression

**Figure 6:**
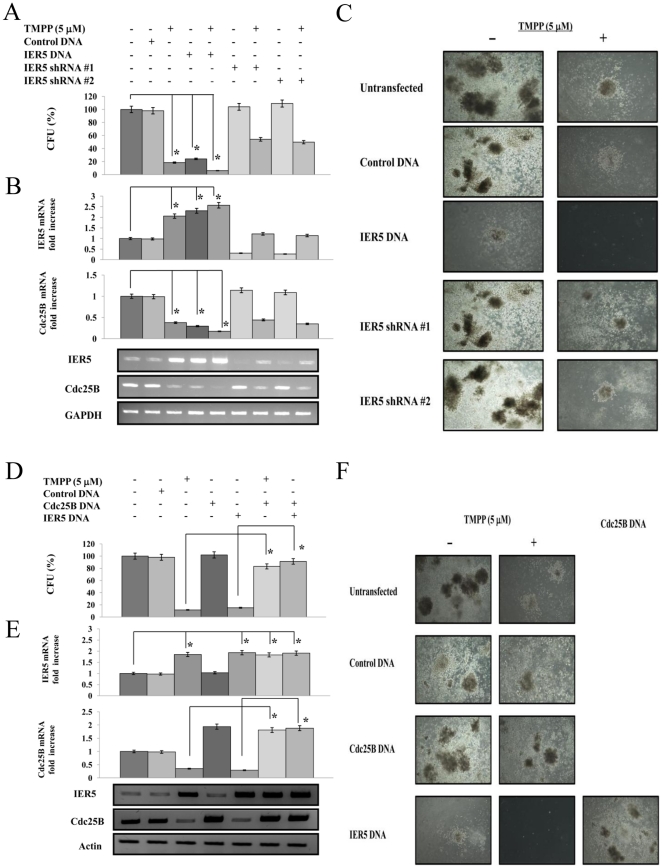
IER5 expression inhibited the colony formation of AML ALDH^hi^/CD34^+^ cells through the regulation of Cdc25B expression. A. ALDH^hi^/CD34^+^ cells were purified from an AML patient (M1) and were cultured in semisolid methylcellulose media. The ALDH^hi^/CD34^+^ cells were either untransfected or were transfected with *IER5* cDNA or with shRNA-#1, or -#2. After 14 days culture with or without TMPP (5 µM), the cells were then analyzed for their colony forming ability. Colony formation of these ALDH^hi^/CD34^+^ cells (3×10^2^ to 5×10^2^ cells/plate) was assessed after plating in semisolid methylcellulose media. B. Analysis of the mRNA expression of *IER5* and *Cdc25B* in each colony using QRT-PCR and RT-PCR. C. The cells were viewed using phase-contrast microscopy after 14 days culture with or without TMPP (5 µM). Original magnification ×4. D. ALDH^hi^/CD34^+^ cells were purified from an AML patient (M1) and were cultured in semisolid methylcellulose media. The ALDH^hi^/CD34^+^ cells were either untransfected or were transfected with *IER5* cDNA or *Cdc25B* cDNA. After 14 days culture with or without TMPP (5 µM), the cells were then analyzed for their colony forming ability. Colony formation of these ALDH^hi^/CD34^+^ cells (3×10^2^ to 5×10^2^ cells/plate) was assessed after plating in semisolid methylcellulose media. Colony formation was evaluated as a percentage of the corresponding control. E. Analysis of the mRNA expression of *IER5* and *Cdc25B* in each colony using QRT-PCR and RT-PCR. The levels of the QRT-PCR products were normalized t*o GAPDH* expression in the same sample and were then expressed relative to the mRNA level of a normal control which was assigned a value of 1. RT-PCR results representative of three independent experiments are shown. *GAPDH* mRNA expression is shown as an internal control. The results are the means ± SD of three independent experiments. *P<0.01 compared with untreated control cells. F. The cells transfected with *Cdc25B* or *IER5* cDNA were viewed using phase-contrast microscopy after 14 days culture with or without TMPP (5 µM). Original magnification ×4.

We examined the effect of induction or knock-down of IER5 on the colony formzing activity of ALDH^hi^/CD34^+^ hematopoietic progenitor cells from an AML patient (M1) (Fig. 6A and C). When cells were left untreated, the mean number of colonies was 117 (range, 102–126). When the cells were transfected with *IER5* cDNA and/or treated with TMPP, the mean numbers of colonies were 7 (range, 3–11), 28 (range, 20–37), and 22 (range, 14–26), respectively. Thus colony numbers were dramatically reduced when ALDH^hi^/CD34^+^ cells were cultured with TMPP and/or were transfected with *IER5 c*DNA. In ALDH^hi^/CD34^+^ cells transfected with *IER5* shRNA-#1 or -#2, TMPP moderately reduced colony numbers compared to untransfected cells. Moreover, in each colony derived from ALDH^hi^/CD34^+^ cells that was treated with TMPP or transfected with *IER5* cDNA, the expression of *IER5* mRNA was increased and the expression of *Cdc25B* mRNA was significantly decreased compared to untreated cells (Fig. 6B). These results demonstrate that during the proliferation of progenitor cells derived from AML patients, IER5 induced a reduction in Cdc25B expression.

To further assess the effects of IER5-mediated Cdc25B reduction on colony formation of ALDH^hi^/CD34^+^ cells from an AML patient (M1), we investigated whether transfection of *Cdc25B* DNA could rescue the colony forming activity of the AML-derived ALDH^hi^/CD34^+^ cells transfected with *IER5* DNA or treated with TMPP. ALDH^hi^/CD34^+^ cells were transfected with *IER5* cDNA or were treated with TMPP. As shown in Fig. 6D and F, either TMPP treatment or IER5 over-expression inhibited colony formation of AML-derived ALDH^hi^/CD34^+^ cells. In contrast, over-expression of Cdc25B in ALDH^hi^/CD34^+^ cells increased the number of colonies compared to untransfected cells. Moreover, the expression level of *IER5* mRNA was not affected in any colony derived from ALDH^hi^/CD34^+^ cells that was transfected with *Cdc25B* DNA (Fig. 6E). Similar results were obtained using AML (M2) ALDH^hi^/CD34^+^ cells. These findings suggest that the transcriptional repression mediated by IER5 regulated Cdc25B expression levels, and, subsequently, decreased the expression of Cdc25B, resulting in inhibition of colony formation.

### The reduction of NF-YB binding to the Cdc25B promoter by IER5 causes the decrease of Cdc25B expression

**Figure 7:**
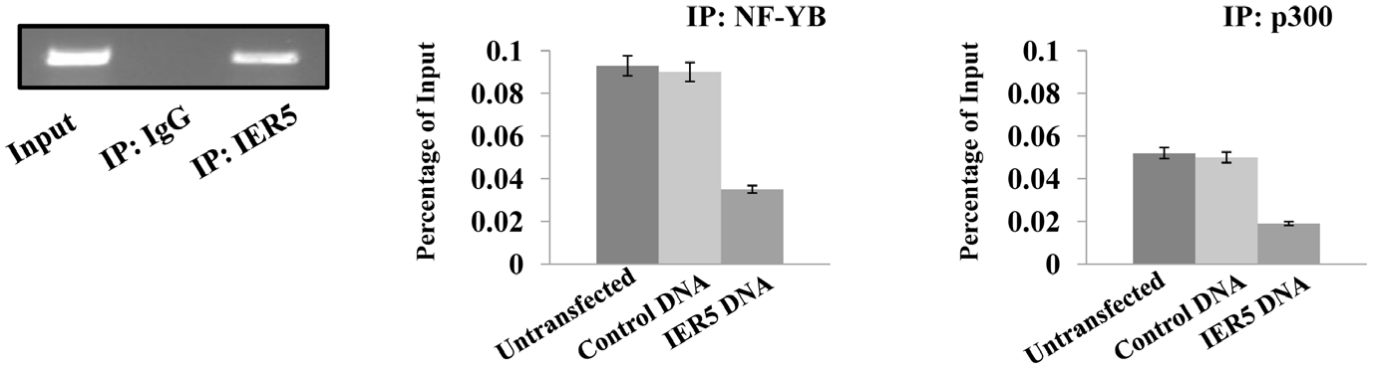
IER5-mediated negative regulation of *Cdc25B* expression through the reduction of NF-YB binding. The ALDH^hi^/CD34^+^ cells purified from two AML patients (AML: M1) were either untransfected or were transfected with control DNA or *IER5* DNA. After 14 days culture, cells were cross-linked with 1% formaldehyde and ChIP was performed with either control antibody (IgG) or the anti-IER5 antibody. The precipitated DNA was then assayed by real-time PCR using pairs (primer set 2) of oligonucleotides encompassing specific region of the *Cdc25B* promoter (left panel). IER5-dependent changes in NF-YB binding (middle panel), and the bindings of coactivators, p300 (right panel) in *Cdc25B* promoter. The values of ChIP efficiencies are given as % of input.

Finally, to confirm binding of IER5 to the *Cdc25B* promoter in AML-derived ALDH^hi^/CD34^+^ cells, we also performed ChIP analysis in ALDH^hi^/CD34^+^ cells purified from one AML patient (AML: M1) (Fig. 7). ChIP analysis showed that IER5 is also highly enriched within a region of the promoter of *Cdc25B* (Fig. 7, left panel). Moreover, to determine whether the over-expression of IER5 corresponded to the binding site of NF-YB within the *Cdc25B* promoter, ChIP analysis was performed using the anti-NF-YB antibody. The result shows that the reduced binding of NF-YB on the *Cdc25b* promoter was observed in IER5 over-expressed AML-derived ALDH^hi^/CD34^+^ cells compared to untreated and control DNA transfected cells (Fig. 7, middle panel). In addition, a significant release of p300 was also observed in IER5 over-expressed AML-derived ALDH^hi^/CD34^+^ cells, (Fig. 7, right panel). These results show that the binding of IER5 on the *Cdc25B* promoter induced the release of the coactivator p300, causing the down-regulation of *Cdc25B* expression in AML-derived ALDH^hi^/CD34^+^ cells.

## Discussion

We investigated the role of IER5 in inhibition of the growth of AML cells induced by treatment with TMPP. We demonstrated, for the first time, that TMPP induces the expression of IER5 and inhibits AML cell proliferation via IER5-mediated transcriptional repression of Cdc25B. Combined with the results of our previous study that TMPP-mediated FOXM1 repression induced G2/M cell cycle arrest, our results provide *in vitro* evidence to support a role for *FOXM1* as an oncogene in AML cells, and to support the fact that its down-regulation inhibits the proliferation of established AML cell lines and primary AML cells.

IER5, which encodes a 308-amino acid member of the immediate-early class of signal transduction proteins, is induced by growth factors and mediates the cellular response to mitogenic signals [6]. IER5 also functions as a transcriptional regulator by binding to DNA or by mediating nuclear protein-protein interactions [6]. Moreover, it has been reported that ionizing radiation induces *IER5* mRNA expression in a dose- and time-dependent manner, and that suppression of IER5 increases the proliferation of HeLa cells and mitigates the inhibition of proliferation induced by exposure to radiation, thereby increasing the resistance of HeLa cells to radiation. In contrast, increased expression of IER5 reportedly disturbed the cell cycle checkpoint and sensitized cells to radiation [11]. These previous results have shown that IER5 expression plays a role in the induction of cell death that is caused by radiation. However, the biochemical pathway and mechanisms of IER5 function in regulation of the cell cycle remain unclear. In this study, we investigated the involvement of IER5 in the cell cycle and in proliferation of AML cells and AML-derived leukemic progenitor cells. We found that treatment with TMPP induced the expression of *IER5* mRNA and that over-expression of IER5 in AML cells inhibited their proliferation. Moreover, suppression of IER5 expression in AML cells using shRNA increased the proliferation of AML cells, and mitigated TMPP-mediated inhibition of proliferation compared to untransfected AML cells. We also found that the both IER5 over-expression and induction of IER5 expression by TMPP treatment induced an increase in the population of AML cells and primary AML blastic cells in the G2/M phase as well as in S phase compared to control untreated cells, 3 days after transfection. These data are consistent with the established notion that IER5 is a key regulator of cell cycle checkpoints [11].

Because induction or over-expression of IER5 expression induced G2/M cell cycle arrest and inhibited AML cell proliferation, we considered that identification of genes regulated by IER5 would facilitate an understanding of the roles IER5 plays in AML cell proliferation. We previously showed that knock-down of FOXM1, or TMPP treatment, markedly decreased the protein expression of the mitotic regulators Skp2, Cdc25B, Cyclin B1, Survivin, KIS, Aurora-B kinase in AML cells [1], [2]. Diminished expression of these mitotic regulators results in a block in mitotic progression or proliferation, and leads to an accumulation of p27*^Kip1^* and p21*^Cip1^* proteins [1], [2]. Moreover, the expression of Cdc25B is known to be increased in AML cells [13]. In this study, we found that IER5 over-expression markedly decreased Cdc25B protein expression, but not the expression of CHK1, WEE1, Cyclin B1, Survivin, and Aurora kinase B proteins. Thus, of the genes in the G2/M specific gene cluster, among which are essential regulators of mitosis, IER5 predominantly controls the expression of Cdc25B. Therefore, IER5 induction would be expected to strongly induce G2/M cell cycle arrest in AML cells. In addition, we observed the number of cells in both S- and G2/M-phase. In cell cycle, the G2/M checkpoint is a barrier to aberrant cellular proliferation, and arrests damaged cells to allow time for repair [17]. The expression of Cdc25B is initiated during S-phase, peaking in G2 and declining in mitosis [18]. Cdc25B has been reported to play a role in the control of entry into mitosis after a DNA damage-activated checkpoint in cells [14], [19], [20]. In AML cells, the Cdc25B participates in the G2/M checkpoint recovery and its expression is upregulated, and the reduction of Cdc25B expression induced the cell number of both S- and G2/M-phase in AML cells [14]. Our data are consistent with the data of the report [14]. Therefore, when TMPP induced the IER5 expression, the Cdc25B expression is reduced and G2/M checkpoint is induced, and then the cells with S- and G2/M-phase DNA contents is increased. Moreover, the population of G2/M distribution is more significantly increased than that of S-phase.

Cdc25 phosphatases, which are dual specificity phosphatases and important regulators of G2/M transition regulators, regulate entry into mitosis by regulating the activation of CDK1/cyclin B [21]. The Cdc25 phosphatases activate the mitotic CDK complexes by dephosphorylating the inhibitory Thr14 and Tyr15 residues on CDK1 and CDK2 [22]. There are three isoforms of the Cdc25 phosphatase; Cdc25A, Cdc25B and Cdc25C, and all three isoforms have roles in G2/M progression. It was previously believed that Cdc25A acts at the G1/S transition, whereas Cdc25B and Cdc25C function mainly at the G2/ M transition [21], [23]. However, recent studies suggest that all three CDC25 phosphatases function as regulators of both G1/S and G2/M transitions [23]. It has also been reported that only depletion of Cdc25A and Cdc25B delays entry into mitosis [24]. In contrast, all three Cdc25 isoforms are targets for checkpoint kinase inactivation [25]. Cdc25A is destabilized by CHK1 phosphorylation in response to DNA damage [26]. Cdc25B is specifically required for exit from the G2 phase checkpoint arrest [27], and its stability has also been linked to responses to damage [28]. Thus, it is believed that aberrant regulation of Cdc25 phosphatases causes tumorigenesis via dysregulation of the cell cycle [22], [29]–[31]. It has been suggested that Cdc25 phosphatases are involved in deficient checkpoints during mitosis in malignant transformation [22], [32]. The activity of the Cdc25 phosphatases is regulated by their phosphorylation status, their expression level and their subcellular localization [22], [32]. Abnormal expression of Cdc25B has been reported in a number of carcinomas, such as non-small cell lung cancer [29], colorectal carcinomas [32], ovarian [33], esophageal [34], [35], prostate [36], gastric [37] and pancreatic [38] cancers. Thus, overexpression of Cdc25B is believed to contribute to tumorigenesis by enhancing tumor malignancy [21]. However, it remains unknown how Cdc25B expression is translationally regulated. Currently, it has been reported that *Cdc25B* is negatively regulated by p53 through Sp1 and NF-Y transcription factors [14]. The analysis of *Cdc25B* promoter shows that the down-regulation of *Cdc25B* by p53 is needed for the Sp1/Sp3 and NF-Y binding sites in *Cdc25B* promoter, p53 binds to the *Cdc25B* promoter and mediates transcriptional attenuation via the Sp1 and NF-Y transcription factors. We have examined the relationship between IER5 and *Cdc25B* promoter, and binding of Sp1 and NF-YB, which is one of NF-Y subunits, by using ChIP assay in AML cell lines and AML-derived ALDH^hi^/CD34^+^ cells. In leukemia cells, IER5 directly binds to *Cdc25B* promoter. Moreover, when IER5 over-expressed, the significantly reduced binding of NF-YB on the *Cdc25B* promoter the release of anti-histone acetyltransferase p300, which is known as a coactivator of NF-Y [15], was observed at upstream of 1st exon of *Cdc25B*. On the other hand, we did not observe that IER5 interacts with Sp1 in AML cells. These results demonstrate that the binding of IER5 on the *Cdc25B* promoter induced the recruitment of coactivator p300, causing the inhibition of AML cell proliferation and colony formation of AML-derived ALDH^hi^/CD34^+^ cells through the down-regulation of *Cdc25B*.

We performed a colony formation assay using the ALDH^hi^/CD34^+^ progenitor cells derived from two normal healthy volunteers and two AML patients (M1 and M2). The number of colonies was moderately reduced when normal ALDH^hi^/CD34^+^ progenitor cells were transfected with *IER5* cDNA. In contrast, colony numbers were significantly reduced when ALDH^hi^/CD34^+^ AML progenitor cells were transfected with *IER5* cDNA or were treated with TMPP. Moreover, the relative expression of Cdc25B was decreased in colony forming cells when ALDH^hi^/CD34^+^ AML progenitor cells were treated with TMPP or were transfected with *IER5* cDNA. Moreover, when ALDH^hi^ /CD34^+^ AML progenitor cells were transfected with *IER5* shRNA, the inhibitory effects of TMPP on colony formation by TMPP were decreased in colony forming cells. These results indicate that increased expression of IER5 induced by TMPP reduced the number of colony forming cells derived from AML progenitor cells through a reduction in Cdc25B expression. Consistent with the idea that IER5 and TMPP effects were mediated by a decrease in Cdc25B, we found that over-expression of Cdc25B rescued both the inhibitory effects of TMPP treatment and those of IER5 over-expression in ALDH^hi^/CD34^+^ AML progenitor cells, resulting in recovery of the colony forming activity of AML-derived ALDH^hi^/CD34^+^ cells.

In conclusion, this study shows for the first time that IER5 over-expression strongly inhibited AML-derived ALDH^hi^/CD34^+^ cell proliferation and colony formation through a reduction in Cdc25B expression, resulting in induction of G2/M cell cycle arrest. Moreover, our data show that the reduction of NF-YB by IER5 inhibited the AML cell proliferation through the release of p300. Therefore, IER5 induction could be an attractive potential target for AML therapy.

## Materials and Methods

### Chemical synthesis and reagents

TMPP was prepared as previously described by reacting 4-bromo-3-methyl-1-phenyl-2-phospholene 1-oxide with bromine [1]. The reaction mixtures were extracted with chloroform, washed with saturated NaCl solution, and dried with anhydrous sodium sulfate. These reaction agents were then dissolved in dimethyl sulfoxide (DMSO) (Sigma-Aldrich, St Louis, MO) and diluted in culture medium immediately before use. The final concentration of DMSO in all experiments was less than 0.01%, and all treatment conditions were compared with vehicle controls. Cytarabine (cytosine arabinoside, Ara-c) was purchased from Sigma-Aldrich.

### Cells and cell cultures

The human leukemia cell lines, KG-1, Kasumi-1 and U937, were purchased from the American Type Culture Collection (ATCC, Manassas, VA). YRK2 cells were harvested in our laboratory from bone marrow samples of AML (French-American-British (FAB) classification; M5a) patients after obtaining informed consent. The KG-1, U937, and YRK2 cells were cultured in RPMI 1640 media containing 10% heat-inactivated fetal bovine serum (FBS), 2 mM L-glutamine, 100 µg/ml streptomycin, and 200 U/ml penicillin (GIBCO-BRL, Gaithersburg, MD). Kasumi-1 cells were grown in RPMI1640 containing 20% FBS, 2 mM L-glutamine, 100 µg/ml streptomycin, and 200 U/ml penicillin. Primary leukemia cell specimens were obtained from the bone marrow of two AML patients (FAB classification; M1 and M2) before the start of any treatment, and normal hematopoietic cells were extracted from healthy donors (n = 2) after obtaining informed consent, which was written. We obtained ethics approval for this study from the Institutional Review Board of Hamamatsu University School of Medicine. Mononuclear cells (MNCs) were purified by Ficoll-Hypaque density-gradient centrifugation.

### Purification of leukemic blast cells based on ALDH activity and CD34 expression

For 2-color staining, MNCs were stained with anti-CD34-phycoerythrin (PE)-conjugated antibody (Becton Dickinson, San Jose, CA) and the Aldefluor reagent (StemCo Biomedical, Durham, NC) according to the manufacturer's specifications and were separated using fluorescence-activated cell sorting (FACS). The ALDH^hi^ cells were gated, and the CD34^+^ cells in the gated ALDH^hi^ population were sorted on a fluorescence-activated cell sorter (Becton Dickinson). Sorted ALDH^hi^/CD34^+^ cell populations were collected in methylcellulose media (Methocut H4435; StemCell Technologies, Inc., Vancouver, BC, Canada).

### RT-PCR and Quantitative Real-Time PCR (QRT-PCR)

Total RNA was extracted from cells using the RNeasy system (Qiagen, Tokyo, Japan), and 2 µg RNA was reverse transcribed using a first strand cDNA synthesis kit (Roche, Indianapolis, IN). PCR was performed using a DNA thermal cycler (model PTC 200; MJ Research, Watertown, MA). The sense and anti-sense oligonucleotide sequences respectively of each primer were as follows: *IER5*, 5′-GGACGACACCGACGAGGAG-3′, and 5′-GCTTTTCCGTAGGAGTCCCG-3′; *Cdc25B*, 5′-TCCAGGGAGAGAAGGTGTCT-3′ and 5′- TGTCCACAAATCCGTCATCT-3′; *GAPDH*, 5′- GAACAGCAACGAGTACCGGGTA-3′ and 5′-CCCATGGCCTTGACCAAGGAG-3′. PCR conditions for *IER5*, *Cdc25B*, and *GAPDH* were: 28 cycles of denaturation at 96°C for 30 sec, annealing at 56°C for 30 sec, and extension at 72°C for 30 sec. All RT-PCR experiments were performed in duplicate. QRT-PCR was performed using SYBER-Green dye and an ABI PRISM 7700 Sequence detector (Perkin-Elmer/Applied Biosystems, Foster City, CA).

### Plasmids and RNA interference

Full-length cDNAs encoding human *IER5* and *Cdc25B* were obtained by RT-PCR using human bone marrow cDNA (BD Biosciences Clontech, Palo Alto, CA) as a template and were cloned into the eukaryotic expression vector pcDNA3.1/V5-His (Invitrogen, Carlsbad, CA). Sequences of recombinant *IER5* and *Cdc25B* cDNAs were verified using automated sequencing.

The vectors for RNA interference (RNAi) specific for human *IER5* were constructed based on the piGENE PUR hU6 vector (iGENE Therapeutics, Tsukuba, Japan), according to the manufacturer's instructions. We used the following targeting sequences: *IER5* shRNA #1; 5′-CCTCATCAGCATCTTCGGT-3′, and *IER5* shRNA #2; 5′-CTGCATAAGAACCTCCTG-3′. The scrambled shRNA sequence, 5′-GGACGAACCTGCTGAGATAT-3′, was used as a control. Vectors were transfected into cells by using the Lipofectamine 2000 kit (Life Technologies, Gaithersburg, MD), according to the manufacturer's instructions. The transfection procedure was repeated 12 h after the first transfection, and cells were harvested at 48 and 72 h after the initial transfection. Knockdown efficiency was consistently 60% to 70%, as determined by RT-PCR measurement of *IER5* mRNA.

### Cell proliferation and viability assay

KG-1, Kasumi-1, U937 and YRK2 cells were transfected with *IER5* shRNA-#1 or -#2, with scrambled shRNA, or were left untreated. After 3 days incubation, the cells were plated in six-well plates at a density of 1×10^4^ cells per well. Cell proliferation was measured by counting cells using a hemocytometer. AML cells were seeded in 96-well, flat-bottom microplates at a density of 2×10^4^ cells per well. The cells were untransfected or were transfected with scrambled shRNA or with *IER5* shRNA-#1 or -#2. Cells grown in complete medium without transfection were used as controls. Cell proliferation was also assessed by counting of viable cells on the indicated days using trypan blue (Sigma-Aldrich) exclusion. The number of nonviable cells was determined by counting of live cells that did not uptake trypan blue in a hemocytometer and are reported as the percentage of untransfected control cells. Each data point was performed in triplicate, and the results are reported as mean counts ± SD.

### Detection of changes in mitochondrial membrane potential (ΔΨm)

To detect Δ*Ψm*, cells (1×10^4^ cells/well) that were transfected with *IER5* cDNA or treated with TMPP were incubated in 24-well plates. After 3 days, cells were labeled with DiOC6 (40 nM in culture medium) at 37°C for 20 min. After washing in PBS, cellular uptake of DiOC6 was analyzed using flow cytometry.

### Cell cycle analysis

Cellular DNA content was analyzed using propidium iodide (PI) (Sigma-Aldrich) staining. The cells were stained with 50 µg/ml PI on day 3 post-transfection. The relative DNA content per cell was measured using flow cytometry and an Epics Elite flow cytometer (Coulter Immunotech, Marseille, France). The percentage of cells in G1, S, and G2/M phases was calculated using the ModFit program (Becton Dickinson, San Jose, CA).

### Western blot analysis

Cells transfected with scrambled shRNA or with *IER5* shRNA-#1 or -#2 were harvested after 3 days. Western blot analysis was performed using the following antibodies: goat polyclonal anti-IER5 (Abcam, Cambridge, UK); Rabbit polyclonal anti-Cdc25B, anti-CHK1, anti-WEE1 and anti-Aurora-B; mouse monoclonal anti-Cyclin B1 and anti-Survivin, all from Santa Cruz. To ensure equal protein loading, Western blotting of actin, used as an internal control, was carried out using a mouse monoclonal anti-Actin antibody (C-4; ICN, Aurora, OH).

### Chromatin immunoprecipitation assay

U937 and AML-derived ALDH^hi^/CD34^+^ cells were untransfected or transfected with control DNA, or *IER5* DNA. After 3 days, the cells were crosslinked with 1% formaldehyde for 15 minutes at room temperature, washed twice with ice-cold phosphate-buffered saline (PBS), and harvested. ChIP assay was performed by using Simple ChIP Enzymatic Chromatin IP Kit (Cell Signaling Technology, Beverly, MA), according to the manufacturer's recommendations. Immunoprecipitated DNA and input DNA were treated with RNase A (Sigma-Aldrich) and proteinase K (Roche), and extracted with phenol:chloroform. For ChIP analysis of IER5, Sp1, Sp3, NF-YB, DNMT1, and p300, anti-IER5 antibody (Abcam), anti-Sp1 (Santa Cruz), anti-Sp3 (Santa Cruz), anti-NF-YB (Santa Cruz), anti-DNMT1 (Abcam), and anti-p300 (Santa Cruz) with protein G-conjugated agarose beads were incubated to make each antibody-conjugated agarose beads. Anti-rabbit IgG antibody was used as an isotype control. DNA that underwent ChIP was analyzed by conventional and quantitative PCR, and data are presented as percentage of input as determined with Applied Biosystem's SDS software Absolute Quantification protocol. Sense and anti-sense oligonucleotide sequences respectively of each primer for PCR were as follows: Set 1 (nucleotide position; -424 to -126), (S1) 5′-AGCCGGGTTGACAGAGGGAGAC-3′, and (AS1) 5′-AACGGTGGAACTAGGAATGGA–3′, Set 2 (nucleotide position; -96 to +120), (S2) 5′- AAGAGCCCATCAGTTCCGCTTG -3′, and (AS2) 5′- CCCATTTTACAGACCTGGACGC -3′.

### Colony forming assays

Colony forming assays were performed by plating purified populations of cells at concentrations ranging from 1×10^3^ to 2×10^3^ cells into methylcellulose media (Methocut H4435; Stem Cell Technologies). Colonies were enumerated under light microscopy (Zeiss, Munchen, Germany) following incubation for 14 days at 37 °C and 5% CO_2_.

### Isolation of progenitor cells and QRT-PCR of progenitor cells

Following colony formation, each colony was harvested using a glass syringe, and all cells of the same colony were pooled and washed. An RNeasy system was used to extract total RNA from approximately 5×10^4^ cells from each colony.

### Statistical analysis

Data are representative of at least three experiments with essentially similar results. These results are expressed as the means ± standard deviations (SD) or standard error of the means as indicated. Statistical analyses of the data were performed using Student's *t*-test. *P* values less than 0.05 were considered statistically significant.
